# Developmental Neurotoxicity Screening for Nanoparticles Using Neuron-Like Cells of Human Umbilical Cord Mesenchymal Stem Cells: Example with Magnetite Nanoparticles

**DOI:** 10.3390/nano10081607

**Published:** 2020-08-15

**Authors:** Teresa Coccini, Patrizia Pignatti, Arsenio Spinillo, Uliana De Simone

**Affiliations:** 1 Toxicology Unit, Laboratory of Clinical and Experimental Toxicology, Istituti Clinici Scientifici Maugeri IRCCS, Via Maugeri 10, 27100 Pavia, Italy; uliana.desimone@icsmaugeri.it; 2 Allergy and Immunology Unit, Istituti Clinici Scientifici Maugeri IRCCS, Via Maugeri 10, 27100 Pavia, Italy; patrizia.pignatti@icsmaugeri.it; 3 Department of Obstetrics and Gynecology, Fondazione IRCCS Policlinico San Matteo and University of Pavia, 27100 Pavia, Italy; spinillo@smatteo.pv.it

**Keywords:** occupational and environmental exposure, risk assessment, human primary cell culture, predictive nanotoxicology, neurotoxicity, Fe_3_O_4_ nanoparticles

## Abstract

Metallic nanoparticles (NPs), as iron oxide NPs, accumulate in organs, cross the blood-brain barrier and placenta, and have the potential to elicit developmental neurotoxicity (DNT). Human stem cell-derived in vitro models may provide more realistic platforms to study NPs effects on neural cells, and to obtain relevant information on the potential for early or late DNT effects in humans. Primary neuronal-like cells (hNLCs) were generated from mesenchymal stem cells derived from human umbilical cord lining and the effects caused by magnetite (Fe_3_O_4_NPs, 1–50 μg/mL) evaluated. Neuronal differentiation process was divided into stages: undifferentiated, early, mid- and fully-differentiated (from day-2 to 8 of induction) based on different neuronal markers and morphological changes over time. Reduction in neuronal differentiation induction after NP exposure was observed associated with NP uptake: β-tubulin III (β-Tub III), microtubule-associated protein 2 (MAP-2), enolase (NSE) and nestin were downregulated (10–40%), starting from 25 μg/mL at the early stage. Effects were exacerbated at higher concentrations and persisted up to 8 days without cell morphology alterations. Adenosine triphosphate (ATP) and caspase-3/7 activity data indicated Fe_3_O_4_NPs-induced cell mortality in a concentration-dependent manner and increases of apoptosis: effects appeared early (from day-3), started at low concentrations (≥5 μg/mL) and persisted. This new human cell-based model allows different stages of hNLCs to be cultured, exposed to NPs/chemicals, and analyzed for different endpoints at early or later developmental stage.

## 1. Introduction

The toxicity of nanoparticles (NPs) is under continuous investigation to understand both the health impacts of atmospheric ultrafine particles and the safer use of engineered nanomaterials. Available evidence reports developmental toxicity of NPs due to their transfer from pregnant body to fetal circulation and offspring body including the developmental neurotoxicity (DNT). Regardless of the exposure route, NPs could reach the blood and translocate to brain. NPs distribution in the bloodstream raises also a particular concern due to the potential NP transfer from placenta to the fetal central nervous system (CNS) and because blood-brain barrier (BBB) develops gradually in the fetal brain this type of direct exposure to NP in utero may have the most damaging consequences [1]. The transplacental diffusion of compounds from the mother to the offspring may be a cause of DNT. Several studies using different placental models, such as rodent and zebrafish (*Danio rerio*) embryogenesis models and perfusion models of the human placenta, demonstrated that NPs (i.e., Au, TiO_2_, SiO_2_, carbon, QD, and polystyrene NPs) can readily cross the placental barrier [2,3,4,5,6,7,8].

The capacity of NPs to pass through the placenta at different gestational stages may rely on NP type, composition, size and surface charge [9]. For example, NPs smaller than 240 nm diameter have been shown to have transplacental activity in an ex vivo human placental perfusion model [10].

A substantial amount of evidence has also demonstrated that NPs can cross the BBB and enter the brain to elicit further cytotoxicity [11,12,13]. For example, metallic NPs can cross the placental barrier and accumulate in the fetal brain causing developmental neurotoxicity on exposure during pregnancy [12]. In animal models, several studies have indicated that the prenatal exposure to NPs caused serious damages on the brain and nervous system [14], underlining the potential vulnerability of the fetal brain to the toxicity of various types of NPs before complete BBB formation.

Although the early evidence suggests that the developing brain may be exposed to NPs and that NP exposure can cause DNT effects, there is still little information on the dose-response relationship between NPs and neurodevelopmental effects [15]. In vitro studies of NP toxicity in culture systems could provide additional mechanistic information that may have particular relevance to in vivo human exposures [16].

Even though standardized procedures for the evaluation of NP toxicity have not yet been defined, the integration of validated in vitro studies into safety assessment strategies is claimed by several institutions and regulatory agencies.

Some in vitro models namely human pheochromocytoma-PC12, rodent microglia BV2 and neurons N27, human microvascular endothelial HMEC, have been used to evaluate potential NP effects on NT and to obtain information on DNT evaluations [15].

Human cell-based models are strongly recommended as relevant alternative methods to reduce the uncertainty in species-specific extrapolation of results and to improve prediction in toxicology. In this respect, the use of stem cells (SCs) currently represents one of the emerging trends in technologies for developing assays and tools. SCs have the advantage, over primary and immortalized cells: (i) to generate large populations of stably-differentiated cells representative of different target species including humans, and (ii) to provide a virgin, nontransformed source of cells which can be differentiated into any lineage and serve as potent in vitro models [17]. In particular, SCs could be used as a potential platform to study the effects of NPs on either undifferentiated or differentiated neural cells, and because SCs can be derived from human tissues, these cultures may provide relevant information on the potential for early or late DNT effects in humans.

It is well documented that SCs with their multi lineage differentiation capacities can be made to differentiate into specific neuronal lineages and will thereby sufficiently mimic cells of the developing brain [18,19]. Successful differentiation of SCs into neuronal lineages is widely reported [20,21,22,23,24]. In particular, recent data confirm that the mesenchymal stem cells derived from human umbilical cord are able to transdifferentiate into neuronal cells and the latter could be used as a powerful tool to assess the DNT in human beings [25,26,27,28,29].

In our recent study, we have reported an established stem cell-based in vitro model system to study various aspects of neurotoxicity (NT) and DNT [30] and proved that human umbilical cord lining-derived stem cells, such as mesenchymal stem cells (hMSCs), when differentiated into neuronal-like cells (hNLCs) may serve as one of the classical tools for the NT and DNT studies.

hMSCs can be easily obtained from the cord lining membranes (CL) of umbilical cord which does not have any ethical concern as placenta is generally discarded after the birth of child and it is obtainable by noninvasive means. In addition, the number of MSCs that can be obtained exceeds the MSCs derived from bone marrow, cord blood, and adipose tissue [31]. Moreover, CL-hMSCs have been proposed as an excellent tool for neurotoxicity testing due to their in vitro differentiation capacity to generate human neuronal-like cells (hNLCs) that may represent a promising source of “health” cells for NT studies. Thus, these hNLCs could provide a new tool for the better understanding of the mechanisms involved in neurotoxicity as well as overcomes the other problems of in vitro data studies by using transformed cell lines. Furthermore, it is possible to reproduce various complex stages of brain development at cellular and molecular level during the differentiation process of hNLCs, making these cells a novel in vitro model for DNT studies/assessment.

Applying this system, the toxicity effects caused by acute exposure (24–48 h) to increasing magnetite nanoparticles (Fe_3_O_4_ NPs) concentrations (10–100 μg/mL) have been demonstrated in our lab using hNLCs at two specific stages of differentiation (i.e., at day 3 and 8). Cell density decrease (20–50%) and apoptotic effects were detected at ≥10 μg/mL in both types of differentiated hNLCs, and remarkably the three-day-differentiated hNLCs appeared more vulnerable than 8-day-differentiated cells [30].

Iron oxide nanoparticle (IONPs), including magnetite (Fe_3_O_4_ NPs), are one of the most versatile nanoparticles which have aroused great interest due to their significant magnetic, chemical, thermal, and mechanical properties [32]. The promising use of IONPs in diagnostic and therapeutic fields and their broad utilization in environmental and occupational setting highlight the potential of such IONPs to reach the brain, making it mandatory the assessment of their possible neurotoxicity [33,34]. Notably, in light of the most recent evidence of the presence of magnetite pollutant nanoparticles detected in the brains (frontal cortex) of people living in contaminated cities, such as Mexico City and Manchester [35], better evaluation systems are urgently required. Airborne magnetite pollution particles (~200 nm in size) can access the brain also directly via the olfactory and/or trigeminal nerves, bypassing the BBB. Given their toxicity, abundance in roadside air, and nanoscale dimensions, traffic-derived magnetite pollution nanoparticles may constitute a chronic and pernicious neurotoxicant, and hence an environmental risk factor for Alzheimer’s disease, for large population numbers globally [36], but even for children due to well recognized special susceptibility of the developing nervous system to environmental toxins [37,38]. Several evidences regarding to methylmercury, lead, alcohol, organophosphates indicate that the nature and severity of the brain function disruption depend on when the exposure occurred and the duration and concentration of neurotoxicants which may result in disorders that can be evident immediately after exposure as well as emerge later in life [39].

Animal experiments are currently the gold standard in DNT testing, however the in vivo approach is argued due to some limitations regarding to ethical concern and lower sensitivity with less predictivity for humans [40]. Novel and alternative models are especially advocated by regulators. In particular, the in vitro neurodevelopmental systems are recommended to elucidate the cellular and molecular mechanism of DNT, to reduce the uncertainty in extrapolation of results, and to improve prediction of human toxicity [41].

In this context, the human primary neurons generated from hMSCs have been set up and the differentiation process divided into stages: undifferentiated, early differentiated, mid differentiated and fully differentiated stages (fully mature neurons). They serve as in vitro model for reproducing various stages of neuronal maturation and studying and monitoring, over time, the brain damage that occurs from early to mature stage. In particular, the present study aimed at evaluating the impact of Fe_3_O_4_NPs exposure on neuron-like differentiation process from CL-hMSCs by applying the challenge (a single treatment) at the beginning of the transdifferentiation process. Different endpoints such as ATP evaluation, cell death and apoptosis, neuronal markers expressions (β-Tub III, MAP-2, NSE, and nestin) and intracellular uptake of NPs during hNLCs differentiation have been evaluated for identifying the adverse effects and molecular mechanisms induced by NPs exposure on nervous system.

## 2. Materials and Methods

### 2.1. Chemicals and Reagents

Mesenchymal stem cell growth medium 2 (Ready-to-use) (PromoCell, Heidelberg, Germany), mesenchymal stem cell neurogenic differentiation medium (Ready-to-use) (PromoCell), human fibronectin solution (1 mg/mL) (PromoCell), and all cell culture reagents were purchased from Carlo Erba Reagents s.r.l. (Cornaredo, Italy). Tissue culture flasks (75 cm^2^; Corning, Amsterdam, The Netherlands) were purchased from VWR International PBI (Italy). Accutase (DUTSCHER) and 96- and 6-well plates (SPL) were purchased from BioSigma (Cona, Italy). CytoTox-Glo™ cytotoxicity, CellTiter-Glo^®^ 3D Cell Viability, and Caspase-Glo^®^ 3/7 assays were acquired from Promega Italia s.r.l (Milan, Italy). Primary antibodies conjugated to alexa-fluo^®^ 488 or 594 for NSE (Santa Cruz Biotechnology, Heidelberg, Germany), and β-Tub III, MAP-2, and nestin (Merck) were purchased from D.B.A. Italia S.R.L. (Segrate, Italy) and Merck (Milan, Italy), respectively. BD cytofix/cytoperm kit was acquired from BD Biosciences (Milan, Italy). Hoechst 33258 was provided by Life Technologies Italia (Monza, Italy). Polyvinylpyrrolidone coated Fe_3_O_4_NPs were obtained from nanoComposix (San Diego, CA, USA; lot no. MGM1837B).

### 2.2. Apparatus

Morphological analysis and Perls’ Prussian blue staining were performed using an inverted microscope (Carl Zeiss, Axiovert 25, Milan, Italy) equipped with a 32X objective combined with a digital camera (Canon Powershot G8, Carl Zeiss, Milan, Italy). CX41 Olympus fluorescence microscope (Olympus Italia S.R.L., Segrate (MI), Italy) equipped with oil immersion objective (100X) lens, combined with a EPI LED Cassette (FRAEN, Settimo Milanese (MI), Italy) for excitation light and with digital camera (Infinity2, FRAEN, Settimo Milanese (MI), Italy), was employed for the fluorescence analysis. Measurement conditions were the following: 470 nm excitation (T% = 40), 505 nm dichroic beamsplitter, and 510 nm long pass filter. Flow cytometry analysis was applied to quantified the neuronal markers expression during the differentiation process using a two-laser flow cytometer (FACSCantoII, BD Biosciences, San Jose, CA, USA) combined with Diva Software. A microcentrifuge PICO 21 Thermo Fisher (Bioidea 2 Sas, Settimo Milanese (MI), Italy) was used to pellet the cells. ATP content, cell death and caspase 3/7 activity were quantified by a microplate fluorometer (Fluoroskan, Thermo Scientific, Milan, Italy) combined with PC software (Ascent 2.6, Thermo Scientific, Milan, Italy).

### 2.3. Human Primary Neuronal Cells: Neuronal Induction from Mesenchymal Stem Cells Derived from Human Umbilical Cord

Samples of umbilical cords were obtained from full-term pregnant women during elective cesarean sections after informed consent (Internal Ethics Committee-Prot. No. 2017000038067, 23 December 2016) gained from each participant healthy donor mothers at the Hospital Fondazione IRCCS Policlinico San Matteo in Pavia, Italy (from January 2017 to January 2019). Human mesenchymal stem cells derived from human umbilical cord lining membranes (CL-hMSCs) were isolated and characterized in our lab as described in Coccini et al. [42]. CL-hMSCs were routinely cultured in 75 cm^2^ flasks using mesenchymal stem cell growth medium 2 and maintained at 37 °C in a humidified atmosphere of 95% air/5% CO_2_.

CL-hMSCs, obtained from n = 4 donors, at passage (P) 3–4, according to the logarithmic growth phase, were induced to transdifferentiation into neuron-like cells using a specific protocol: CL-hMSCs were cultured in standard conditions until ~80% cell confluence was reached, then the cells were detached by Accutase and reseeded on multiwell plates (at 4000 or 1500 cells/cm^2^ for 6- and 96-well cell culture plate, respectively) coated with 10 μg/mL human fibronectin in mesenchymal stem cell growth medium 2. After 72 h, the whole culture medium (mesenchymal stem cell growth medium 2) was replaced with a ready-to-use mesenchymal stem cell neurogenic differentiation medium. The medium was changed every 48 h. hNLCs were harvested at different stages of differentiation (early-, mid- and fully differentiated-stage), specifically the following time points were considered: after 2 and 3 (early), 4 and 5 (mid) and 8 (fully) days. One hour after induction (undifferentiated stage) was also considered for the morphological analysis. Morphological changes were monitored over time (up to 8 days) using an inverted phase-contrast microscope.

### 2.4. Fe_3_O_4_NPs Stock Suspension

The physico-chemical properties of Fe_3_O_4_NPs stock suspension in a 2 mM citrate solution were provided by the nanoComposix Company and summarized in Table 1.

**Figure 1:**
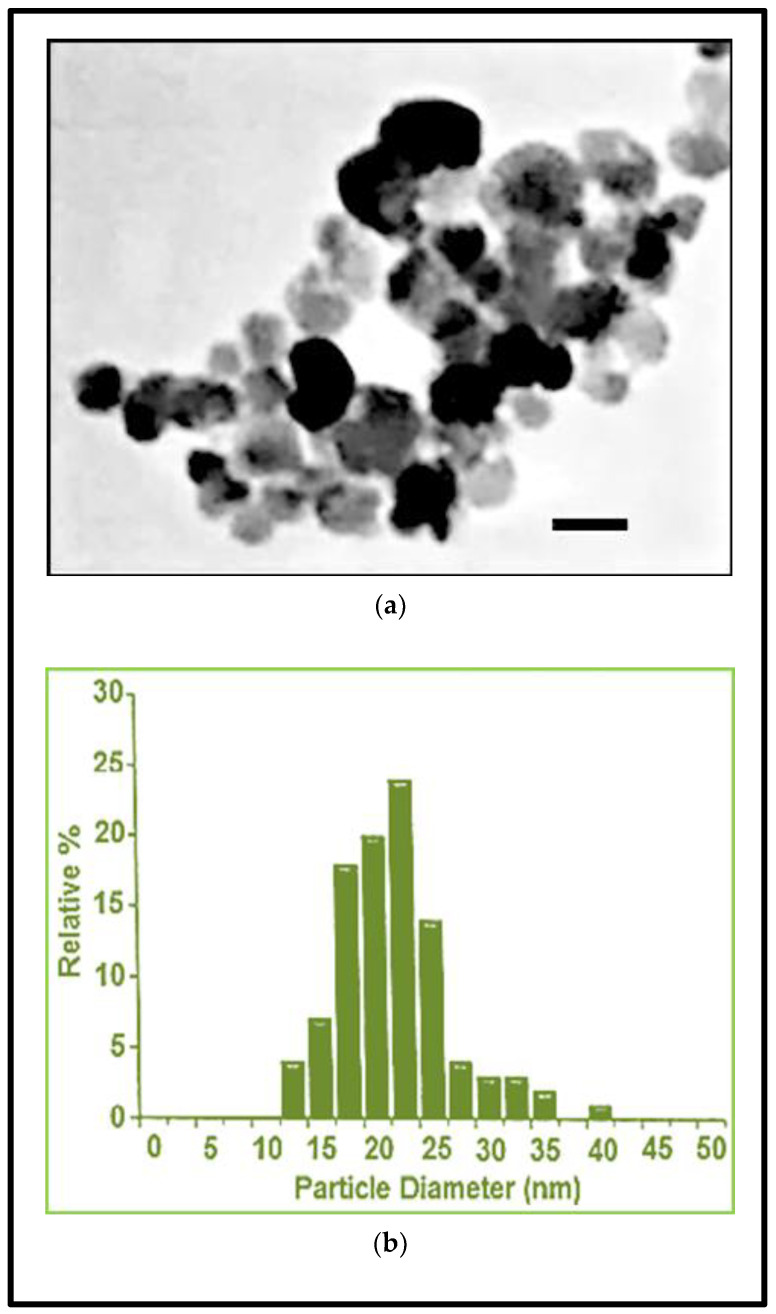
Fe_3_O_4_NPs stock suspension. (**a**) Morpho-dimensional analysis by transmission electron microscopy. Scale bar: 20 nm; (**b**) hydrodynamic diameter evaluation by dynamic light scattering measurements (data provided by nanoComposix Company).

The surface of Fe_3_O_4_NPs was functionalized with polyvinylpyrrolidone, a polymer that offers steric stability and exhibit superparamagnetic properties at ambient temperatures. Morpho-dimensional analysis using transmission electron microscopy showed that Fe_3_O_4_NPs, dark brown in color, had a roughly spherical, almost non-agglomerated particles, an average diameter of 20.3 ± 5 nm (by transmission electron microscopy) and a hydrodynamic diameter of 42 nm (by dynamic light scattering measurements) (Figure 1a,b).

Physico-chemical Fe_3_O_4_NPs properties in mesenchymal stem cell neurogenic differentiation medium (Table 2) were performed as detailed in De Simone et al. [30]. The parameters were evaluated up to 48 h since fresh neurogenic medium was changed every 48 h.

### 2.5. Fe_3_O_4_NPs Exposure

Exposure to increasing concentrations of Fe_3_O_4_NPs (from 1 to 50 μg/mL) was performed at the beginning of the transdifferentiation process of CL-hMSCs into hNLCs, namely T = 0. In particular, CL-hMSCs were cultured as described above in mesenchymal stem cell growth medium 2 for 72 h (~80% cell confluence was reached) then culture medium was aspirated and replaced with neurogenic differentiation medium containing Fe_3_O_4_NPs (T = 0). Different Fe_3_O_4_NPs concentrations (from 1 to 50 μg/mL) were evaluated. Fresh neurogenic medium (without Fe_3_O_4_NPs) was changed every 48 h up to 8 days, and hNLCs were harvested at different stages of neuron-like cells maturation (early-, mid- and fully-differentiated-stage), specifically the following time points were considered: after 2, 3, 4, 5 and 8 days. Multiple endpoints of toxicity in term of morphological changes, expression of neuronal markers, cell viability, ATP content and caspase 3/7 activity were evaluated at each time point. One non-induced culture dish was also analyzed in every experiment as negative control. The cells were daily monitored after neuronal induction.

### 2.6. Intracellular Fe_3_O_4_NPs Detected by Perls’ Prussian Blue Staining

To visualize the intracellular iron in hNLCs, at day 3 and 8 of transdifferentiation process treated with single concentrations of Fe_3_O_4_NPs (1–50 μg/mL), Perls’ Prussian blue staining was performed. Briefly, culture medium was removed and hNLCs were washed twice with PBS and fixed (4% paraformaldehyde solution in PBS (PF), 20 min at room temperature. Subsequently, the cells were washed twice with PBS then incubated with Perls’ solution (1:1 solution 2% of K_4_[Fe(CN)_6_] and solution 6% HCl; 2 mL/well) for 30 min at room temperature. After washing three times with deionized water, hNLCs were counterstained with 0.5% neutral red solution up to 1 min, washed and let dry. hNLCs (at day 3 and 8) were examined under an inverted microscopy.

### 2.7. Neuronal Induction Evaluation

#### 2.7.1. Morphological Analysis by Light Phase-Contrast Microscopy

The hNLCs at different stages of differentiation (i.e., 2, 3, 4, 5, and 8 days) were observed under an inverted phase-contrast microscopy in order to analyze the healthy status of the cells, their growth and the morphological changes induced by the transdifferentiation process as well as after Fe_3_O_4_NP treatments.

#### 2.7.2. Neuronal Markers

##### Expression by Immunofluorescence Analysis

Expression of neuronal markers by immunofluorescence analysis was performed during the neuronal differentiation process (i.e., 2, 3, 4, 5, and 8 days after induction) without Fe_3_O_4_NP treatments as well as in hNLCs treated with Fe_3_O_4_NP (10–50 μg/mL) at day 3 and 8. In particular, hNLCs were rinsed with PBS gently and fixed in 4% PF (30 min at room temperature), then permeabilized with 0.1% Triton-100 for 5 min. Subsequently, the neuron-like cells were washed (three times with PBS) and blocked in PBS containing 2% dry milk (30 min at room temperature), followed by incubation for 60 min (at room temperature) with alexa-fluor^®^488 or 594 conjugated primary antibodies against: β-Tub III (1:100), MAP-2 (1:100) and NSE (1:100). After washing in PBS (three times; 5 min) the nuclei were detected using Hoechst 33258 (5 μM for 10 min at room temperature) and finally hNLCs were mounted with Fluoroshield. Fluorescence images were acquired using a fluorescence microscope, and analyzed by imageJ software 1.51 (NIH, Massachusetts, USA) to quantify the fluorescence intensity.

##### Expression by Flow Cytometry Analysis

Flow cytometry analysis was also performed to quantify the changes of the neuronal markers expression both during the neuronal maturation process (up to 8 days) and in hNLCs (at day 3 and 8) treated with Fe_3_O_4_NPs. The cells were washed and detached by incubating them for 5 min at room temperature with Accutase. Thereafter, two volume of PBS containing 2% fetal bovine serum were added and the cells were collected and counted using the Burker chamber to determine the cell viability. Subsequently, hNLCs (at different time points) were diluted to obtain a density of 10^5^ cells for the analysis. Centrifugation steps were conducted using a microcentrifuge at 2400 rpm for 3 min Afterword, the cells were fixed and permeabilized using the BD cytofix/cytoperm kit according to the manufacturer’s instructions. In brief, following the centrifugation, the pellet was re-suspended in Fixation/Permeabilization solution then incubated in the dark for 20 min at +4 °C. Cell pellets were then washed twice with Perm/wash buffer 1× and centrifuged. At this point the cell pellets were re-suspended and incubated with the alexa-fluor^®^488 or 594 conjugated primary antibodies against: β-Tub III (1:10000), MAP-2 (1:200), NSE (1:50), and nestin (1:500) diluted in Perm/wash buffer 1× for 30 min at +4 °C. Then, the cells were washed as described above and cell pellets resuspended in PBS containing 0.5% bovine serum albumin for the analysis. Samples were acquired with a two-laser flow cytometer and the values were expressed as median fluorescence intensity (MFI).

### 2.8. ATP Evaluation by CellTiter-Glo^®^ 3D Assay

The CellTiter-Glo^®^ Luminescent Cell Viability Assay is a luminescence-based test method of determining the number of viable cells in culture based on quantitation of the ATP present, an indicator of metabolically active cells. The ready-to-use solution induces cell lysis and generation of a luminescent signal which is proportional to the amount of ATP present. In according to the protocol supplied by the manufacturer, the ATP content was evaluated and quantified by measuring the luminescence signal using a microplate fluorometer. Background luminescence (blank) associated with the specific cell culture medium and reagent was also determined. The experimental values were obtained by subtracting the blank value.

### 2.9. Cytotoxicity Assay (Dead Cells)

The cell death/viability of hNLCs was determined using the CytoTox-Glo Cytotoxicity Assay () a luminescent assay that allows measurement of the relative number of dead cells in cell population. The assay uses a luminogenic peptide substrate (alanyl-alanyl-phenylalanyl-aminoluciferin: AAF-Glo™ Substrate) to measure “dead-cell protease activity”, which is released from cells that have lost membrane integrity: the AAF-Glo™ substrate cannot cross the intact membrane of live cells and does not generate any appreciable signal from the live-cell population. The liberated aminoluciferin product is measured as “glow type” luminescence generated by Ultra-Glo™ Recombinant Luciferase provided in the assay reagent. The assay selectively detects dead cells. The cell viability was determined following the manufacturer’s instructions. The first step allowed to determine the luminescence signal derived from dead cells, and the second step, after adding the lysing reagent, allowed to measure the total cells (live plus dead). The luminescence signal derived from both steps was quantified using a microplate fluorometer. The background luminescence associated with the specific cell culture medium and assay reagents was evaluated and subtracted from experimental values. The cell viability was calculated with the following formula:

Viable cell Luminescence = Total cell luminescence − Dead cell luminescence(1)

and the cell viability was expressed as the percentage relative to the untreated control cells.

### 2.10. Caspase Activity

Caspase-Glo^®^ 3/7 assay is a luminescence-based test system that measures the caspase-3 and -7 activities. Adding caspase-Glo^®^ 3/7 Reagent in an “add-mix-measure” format results in cell lysis followed by caspase cleavage of the substrate and generation of a luminescent signal, produced by luciferase, which is proportional to the amount of caspase activity present. In according to the protocol supplied by the manufacturer the Caspase-Glo^®^ 3/7 activity was evaluated in hNLCs (at day 2, 3, 4, 5, 8) and the luminescence signal quantified using a microplate fluorometer. Background luminescence (blank) associated with the culture medium used for hNLCs was determined. Then, the experimental values were obtained by subtracting the blank value.

### 2.11. Statistical Analysis

Data of the flow cytometric analysis, ATP content, cell viability, caspase-3/7 activity are shown as the mean ± S.D. of three separate experiments, each carried out in three or four replicates. Statistical analysis was performed by two-way ANOVA followed by Dunnett’s test. Only *p*-values less than 0.05 were considered to be significant.

## 3. Results

### 3.1. Morphological Changes over Time after Neuronal Induction

Mesenchymal stem cell neurogenic potential was firstly assessed and then the effects caused by Fe_3_O_4_NP exposure at T0 of the differentiation process were evaluated.

#### 3.1.1. hNLCs Untreated

**Figure 2:**
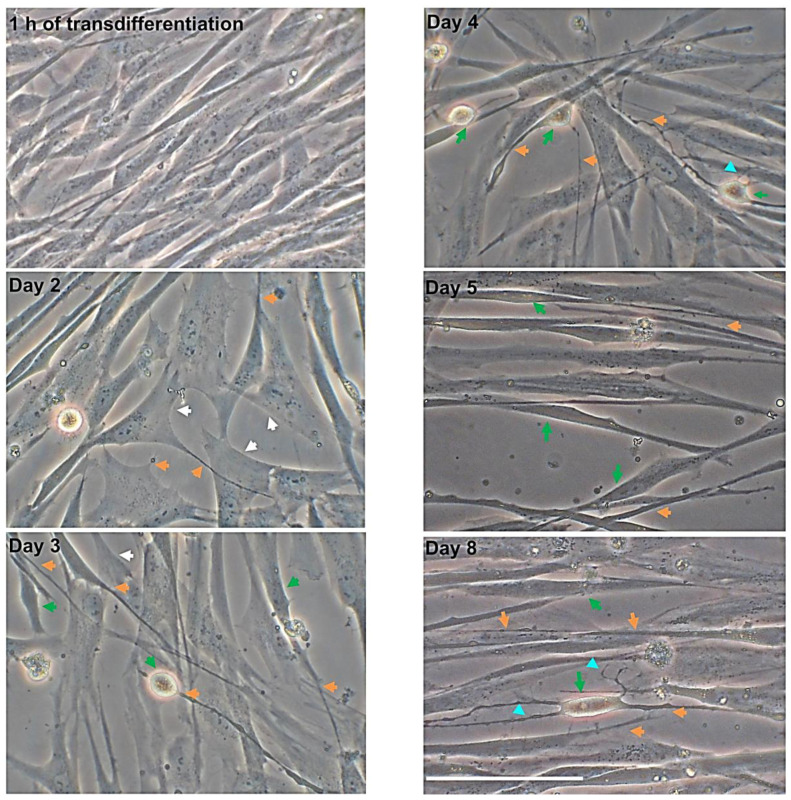
Morphological analysis of CL-hMSCs during transdifferentiation into neuronal-like cells over time. Representative sequence of phase-contrast photomicrographs of hNLCs at 1 h, 2, 3, 4, 5 and 8 days after the transdifferentiation of CL-hMSCs using neurogenic medium. Morphological changes were observed at different stages of hNLCs: the cells adopted a neural-like morphology ranging from spindle shaped (after 1 h transdifferentiation) to spherical with retractile cell bodies (from day 3 to day 5) and long branching processes (day 8, differentiated-stage). Pyramidal cell body and cytoplasm retracted are indicated by white and green arrows respectively, elongated structures are indicated by orange arrows, long extensions with secondary branches are indicated by turquoise headed. Scale bar: 100 μm.

To assess hMSCs neurogenical potential, the morphological changes over time were analyzed in MSCs transdifferentiated into neurons. Following eight days in neural induction medium, MSCs from CL changed their morphology from flat, spindle-shaped to neural-like cells which included retraction of the cytoplasm towards the nucleus with several cytoplasmic extensions (Figure 2).

Specifically, one hour after starting the transdifferentiation induction, the morphological change versus a neuronal phenotype was not yet evident. CL-hMSCs still showed the characteristic spindle-shaped morphology and formed a homogeneous monolayer typically belonging to mesenchymal stem cells. Changes in CL-hMSCs morphology were evident on day 2: CL-hMSCs lost their characteristic spindle-shaped fibroblastic morphology and began to exhibit a neural-like morphology and displayed a pyramidal cell body with retraction of cytoplasm and process-like extensions peripherally. After 3 days of induction, the morphological changes observed on day 2 became more evident: cytoplasm retracted towards the nucleus and cells displayed long and thin processes that exhibited simple bipolar expansion that formed a network with adjacent cells. On day 4, hNLCs continued in their transdifferentiation process, many cells had neuronal-like phenotype: cytoplasm retracted and long bipolar expansions from cell bodies were visible. After five days of transdifferentiation, the changes were more distinguishable when compared to three and four days of neural transdifferentiation (cytoplasm retraction and long bipolar cellular processes). On day eight, the majority of the cells showed highly retracted and transparent bodies, and developed long and thin cellular processes some of which formed secondary branches. More extensive network was also observed (Figure 2).

#### 3.1.2. hNLCs Exposed to Fe_3_O_4_NPs

**Figure 3:**
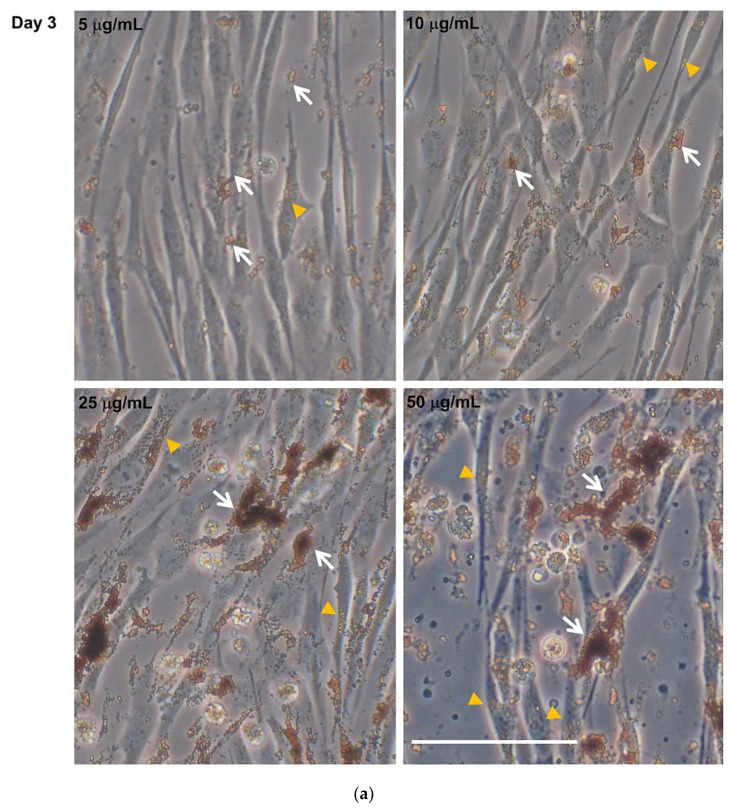

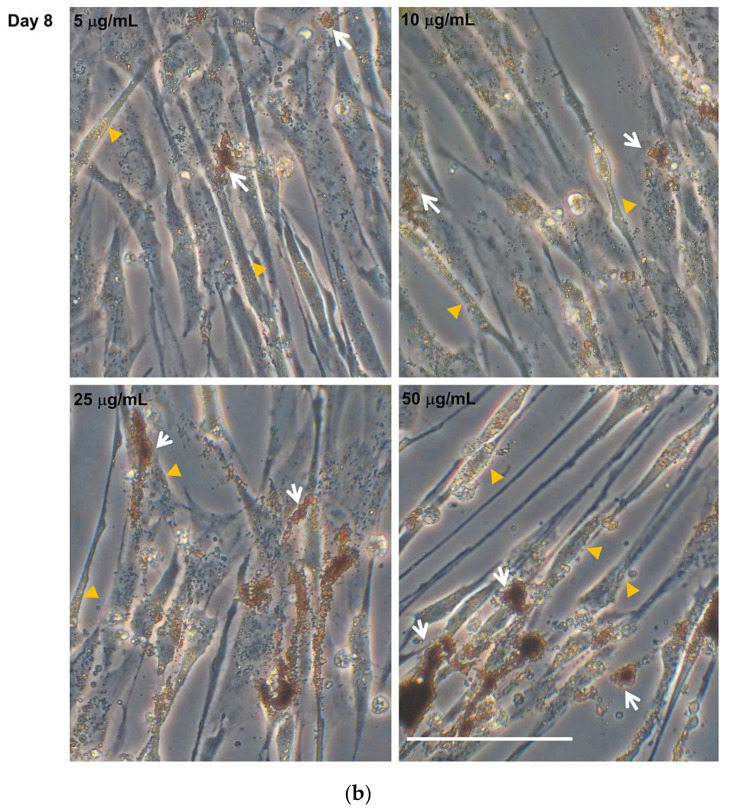
Morphological analysis of hNLCs cultured with Fe_3_O_4_NPs. Representative micrographs, by phase-contrast microscopy, of hNLCs after 3 (**a**) and 8 (**b**) days of culture in neurogenic medium with increasing Fe_3_O_4_NPs concentrations (5–50 μg/mL). No morphological changes were induced by Fe_3_O_4_NPs exposure in hNLCs at day 3 and 8 of transdifferentiation. However, a cell density decrease was observed from 25 μg/mL Fe_3_O_4_NPs at both time points considered. Brownish aggregates/agglomerates of Fe_3_O_4_NPs were observed in culture medium and intracellularly. White arrows indicate Fe_3_O_4_NP aggregates/agglomerates, orange heads indicate intracellular nanoparticles. Scale bar: 100 μm.

In cells exposed to different concentrations of Fe_3_O_4_NPs (single treatment), applied at T0 of the differentiation process, no morphological changes were detected in hNLCs during differentiation process up to eight days; but a cell density decrease was evidenced, by inverted phase-contrast microscopy, starting at 25 μg/mL (Figure 3a,b).

Notably, a spontaneous Fe_3_O_4_NPs uptake in the cytoplasm of the cell body was observed by light-phase contrast microscopy as also highlighted by Perls’ Prussian blue staining (see intracellular Fe_3_O_4_ nanoparticle evaluation by Perls’ Prussian blue staining in the next paragraph). The trend of Fe_3_O_4_NPs uptake was similar at day 3 and 8 of transdifferentiation. NPs were also visible over the cell membranes. Moreover, Fe_3_O_4_NPs appeared finely dispersed in culture medium, and fine aggregates/agglomerates were visible starting from 1 μg/mL which became larger at higher concentrations and persisted even when the culture medium was replaced to keep the cells healthy (once and four times for 3 and 8 day hNLCs, respectively) (Figure 3a,b and Figure S1). In particular, as showed in Table 2, hydrodynamic size of Fe_3_O_4_NPs indicated that Fe_3_O_4_NPs agglomerated/aggregated immediately after dispersion in the culture medium exhibiting a diameter on the order of the micron range: 1213 ± 23.5 and 1368 ± 10 nm at 10 and 25 μg/mL, respectively, after 30 min. The aggregation still persisted after 24 and 48 h (diameter from about 1300 to 1500 nm).

### 3.2. Intracellular Fe_3_O_4_ Nanoparticle Evaluation by Perls’ Prussian Blue Staining

**Figure 4:**
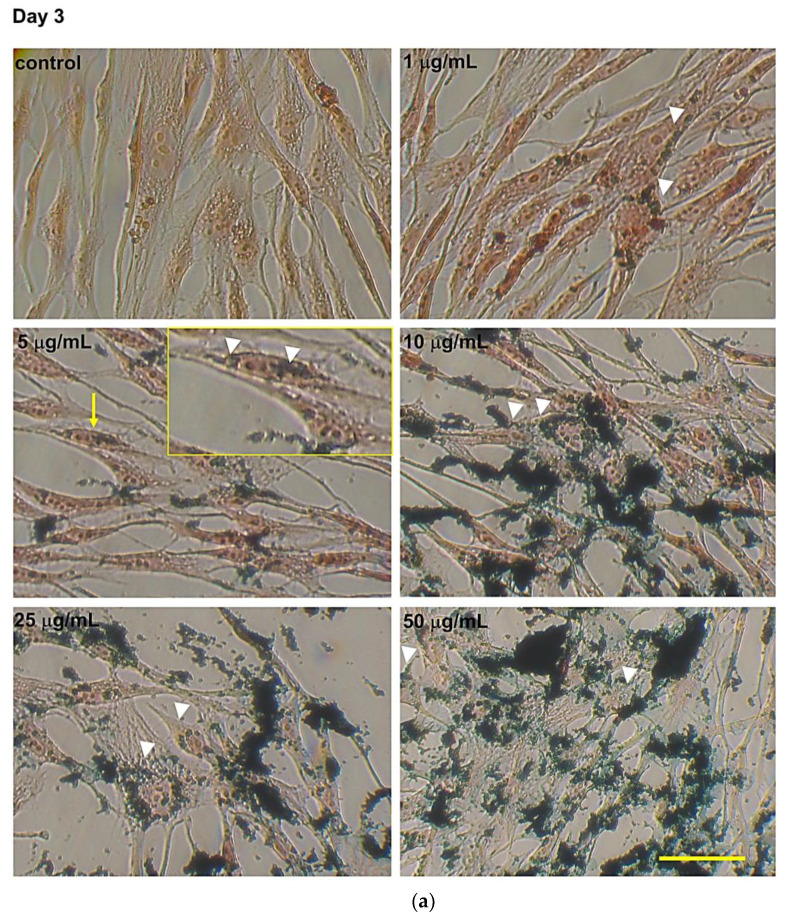

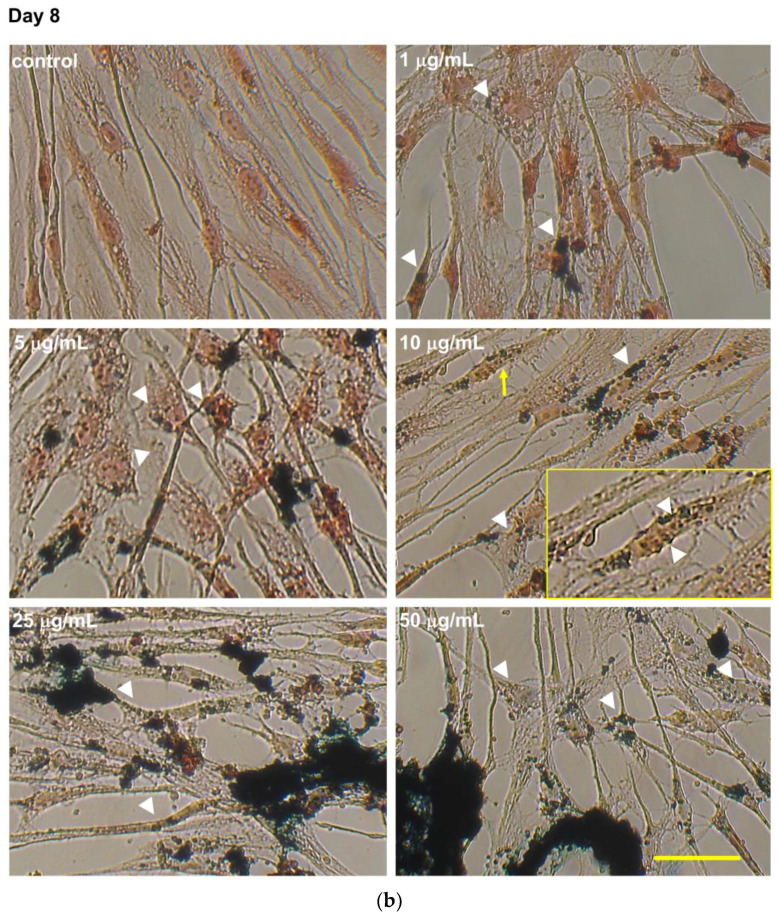
Intracellular evaluation of Fe_3_O_4_NPs by Perls’ Prussian blue staining in hNLCs cultured with Fe_3_O_4_NPs. Representative micrographs, by light microscopy, of hNLCs after 3 days (**a**) and 8 days (**b**) in neurogenic medium with increasing concentrations of Fe_3_O_4_NPs (1–50 μg/mL). Prussian blue staining showed fine intracellular blue spots of Fe_3_O_4_NPs around the nucleus already at the lowest concentration tested (1 μg/mL). The intracellular iron accumulation increased with increasing concentration and the aggregations/agglomerations of Fe_3_O_4_NPs were also visible extracellularly. White heads indicate intracellular nanoparticles and yellow arrows indicate the magnifications (2X) of the areas of the insert selection. Scale bar: 100 μm.

A concentration-dependent Fe_3_O_4_NPs accumulation was evidenced in hNLCs by Perls’ Prussian blue staining. In particular, at day three (Figure 4a) and eight (Figure 4b) of the transdifferentiation, fine intracellular blue spots were visible starting at the lowest concentration tested (1 μg/mL) which enlarged at increasing concentrations (≥5 μg/mL). The intracellular Fe_3_O_4_NPs were mainly found in the cytoplasm region (see inserts in Figure 4a,b), but not in the cell nucleus. Fe_3_O_4_NPs were also evident, extracellularly, attached on the cell membrane or attached on the bottom of the culture well. Notably, a decrease of the extracellular Fe_3_O_4_NPs was observed over time (day 8 versus day 3) probably due to culture medium replacement for keeping cells healthy. Negative controls such as untreated-hNLCs at different stages of transdifferentiation did not have blue dots.

### 3.3. Expression of Neural Markers

**Figure 5:**
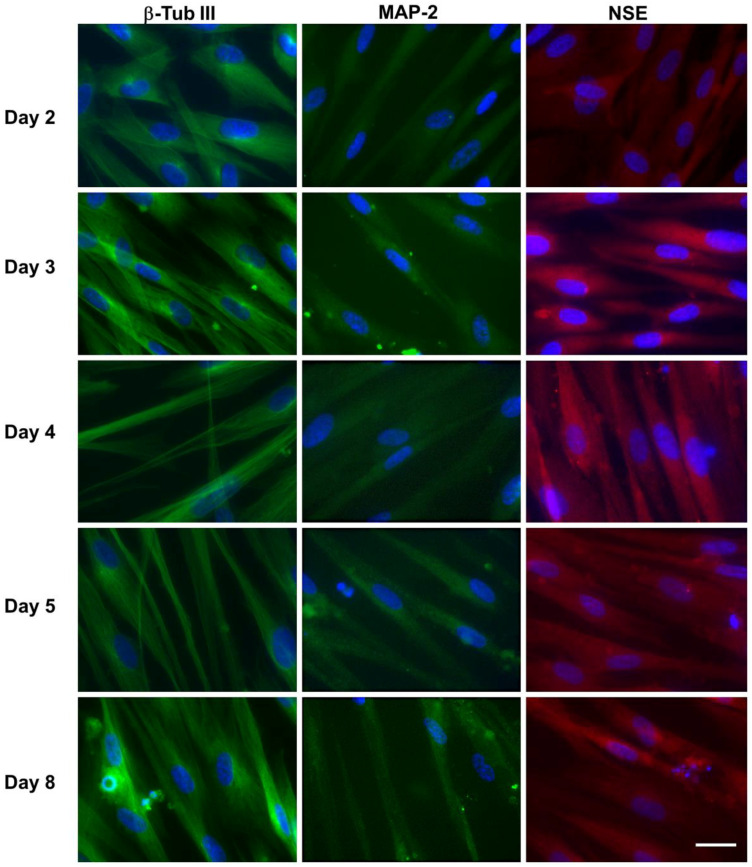
Immunofluorescence analysis of the neuronal marker expression in hNLCs at different time points of transdifferentiation. Representative fluorescence merged microphotographs showing β-Tub III (structural marker), MAP-2 (mature neuron marker) and NSE (cytoplasmic protein expressed by mature neuron) in transdifferentiated hNLCs from day 2 to 8. The fluorescence intensity of β-Tub III (green fluorescence), MAP-2 (green fluorescence) and NSE (red fluorescence) increased in parallel with the progression of neuron-like differentiation of CL-hMSCs cultured in neurogenic medium. Nuclei were stained with Hoechst 33258. Scale bar: 100 μm.

**Figure 6:**
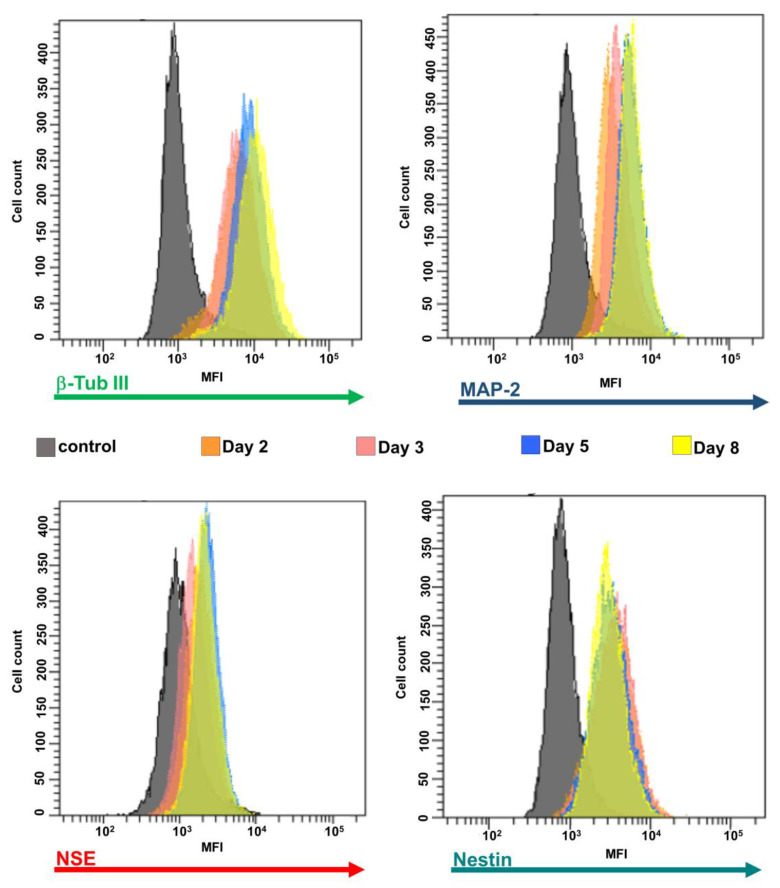
Expression of the neuronal markers during differentiation process. A typical flow cytometry experiment with neuronal markers and hNCLs. Control cells (without antibodies) are shown as filled gray profiles and cells incubated with alexa-fluor^®^488 or 594 labelling for β-Tub III, MAP-2, NSE, or nestin are each indicated as different color profiles at the different stages of differentiation (i.e., 2, 3, 5, and 8 days).

The differentiation status of hNLCs from CL-hMSCs was also verified through the expression of different neuronal markers such as β-Tub III, MAP-2, NSE, and nestin by immunocytochemistry staining (Figure 5) and flow cytometry (Figure 6).

#### 3.3.1. hNLCs Untreated

##### Evaluation by Immunofluorescence Staining

The results of immunofluoresence analysis confirmed the expression of β-Tub III (structural marker expressed exclusively by neurons), MAP-2 (mature neuron marker), and NSE (cytoplasmic protein expressed by mature neuron) in transdifferentiated hNLCs from day 2 to 8. The fluorescence intensity of β-Tub III (green fluorescence around the soma and neurite-like processes), MAP-2 (green fluorescence into the cytoplasm and neurite-like processes), and NSE (red fluorescent signal into cytoplasm) increased in parallel with the progression of neuron-like differentiation of CL-hMSCs cultured in neurogenic medium (Figure 5). In particular, the expressions of all these markers were more elevated in hNLCs at day 8-fully differentiated than early (day 2 and 3) and mid (day 4 and 5) stages of transdifferentiation (Figure 5).

##### Evaluation by Flow Cytometry

Flow cytometric analysis revealed that the β-Tub III, MAP-2 and NSE increased from day 2 to day 8 as shown by the MFI shift in Figure 6. Differently, nestin MFI decreased over time (Figure 6). Quantitative analysis of neural markers through the MFI of β-Tub III in neural induced cells derived from CL-hMSCs indicated an increase by 57% from day 2 of the induction process to day 8 (MFI value at day 2 was 5600 ± 310); the MFI of MAP-2 also gradually increased over time reaching 95% at day 8 (MFI value at day 2 was 2110 ± 250); and the MFI of NSE ranged in untreated cells from 810 to 1600 (Figure 6 and Figure 8). The evaluation of the differentiation of the nestin, a protein marker for neural stem cells, indicated an early presence and later downregulation: MFI was high at the early time point (day 2) and decreased to about 44% with the neuronal differentiation (on day 8) (MFI value at day 2 was 2760 ± 500) ((Figure 6 and Figure 8).

#### 3.3.2. hNLCs Exposed to Fe_3_O_4_NPs

##### Evaluation by Immunofluorescence Staining

**Figure 7:**
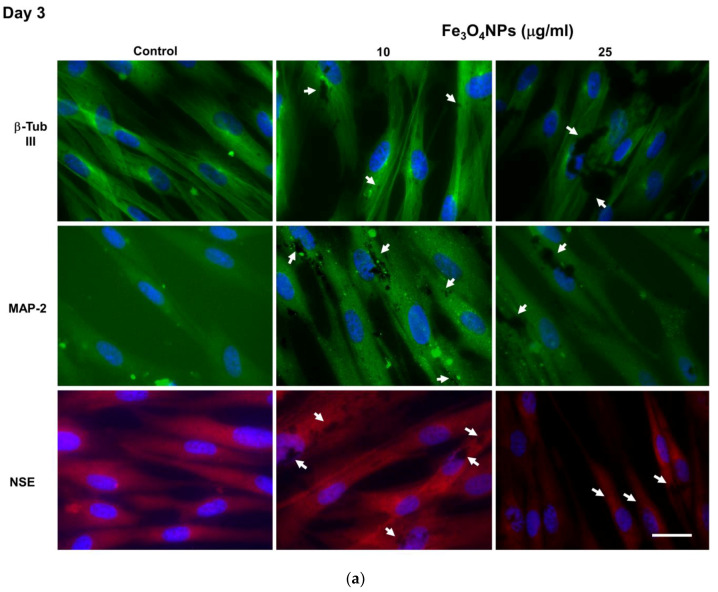

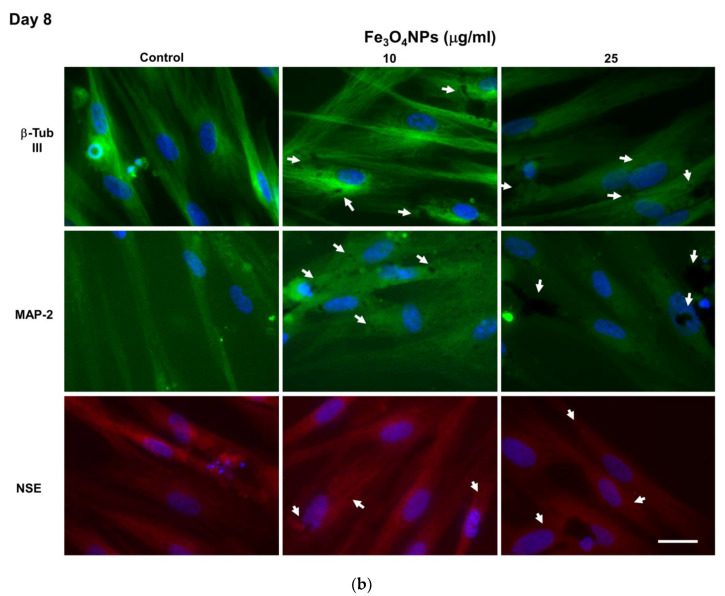
Neuronal marker protein expressions in hNLCs cultured with Fe_3_O_4_NPs. Fe_3_O_4_NPs apparently affected the transdifferentiation of CL-hMSCs starting from 25 μg/mL: hNLCs at day 3 (**a**) and day 8 (**b**) of transdifferentiation exhibited a decrease of fluorescence intensity of neuron markers such as β-Tub III (green fluorescence), MAP-2 (green fluorescence) and NSE (red fluorescence). Black spots of Fe_3_O_4_NPs (intracellularly and on the cell membrane) were also visible. Nuclei were stained with Hoechst 33258. White arrows indicate Fe_3_O_4_NPs (dark spots). Scale bar: 100 μm.

Fe_3_O_4_NPs treatments apparently affected the transdifferentiation of CL-hMSCs when evaluated by immunofluorescence microscopy in that a decrease of the fluorescence intensity was observed starting from 25 μg/mL: neuronal markers such as β-Tub III, MAP-2, and NSE were visible as green fluorescence around the soma and neurite-like processes in hNLCs at both time points (day 3 and day 8) of the neurogenic transdifferentiation, and NSE was visualized as red fluorescent signal into cytoplasm (Figure 7a,b and Figure S2). The reduction was about 35–45% for β-Tub III, 30–35% for MAP-2 and 25–30% for NSE. Black spots of Fe_3_O_4_NPs, both intracellularly and on the membrane were also visible in hNLCs at day 3 and 8.

##### Evaluation by Flow Cytometry

**Figure 8:**
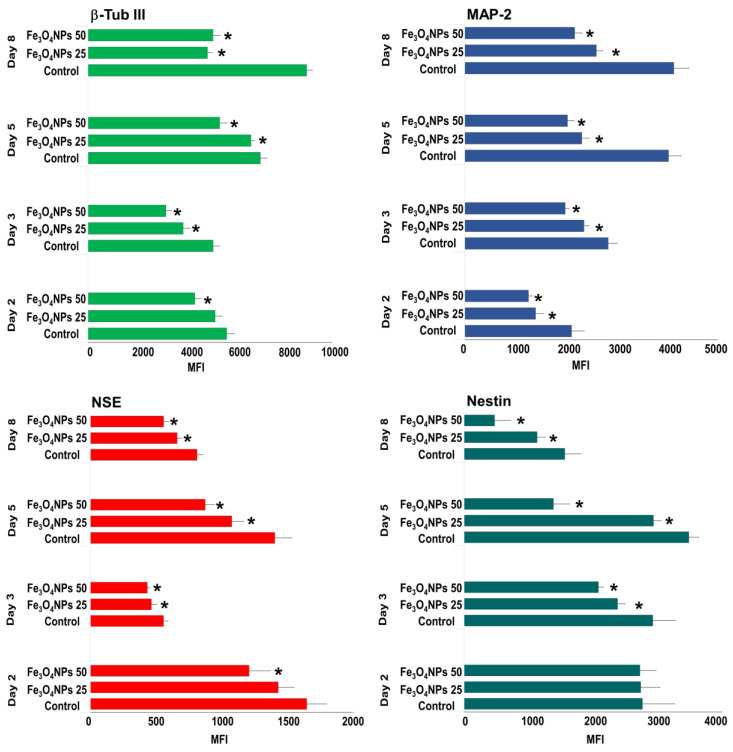
Flow cytometric analysis of the expression of neuronal markers over time in hNCLs untreated and exposed to Fe_3_O_4_NPs. Data are expressed as MFI and represent the mean ± S.D. * *p* < 0.05, statistical analysis by two-way ANOVA followed by Dunnett’s test.

The evaluation by flow cytometry indicated that Fe_3_O_4_NPs exposure significantly downregulated the β-Tub III, MAP-2, NSE differentiation proteins in a concentration-dependent manner, starting from 25 μg/mL and exacerbating at 50 μg/mL. These effects appeared early (day 2) and persisted up to day 8 (Figure 8). While, 10 μg/mL did not cause modification (data not shown). Even the nestin was downregulated when Fe_3_O_4_NPs were added at the beginning of the differentiation process. In details, a significant decrease (*p* < 0.05) of these neuronal proteins, from day 2 to 8, were determined. At 25 μg/mL, the decrease ranged from 10 to 45% for β-Tub III; from 16 to 43% for MAP-2, from 13 to 23% for NSE, and from 15 to 27% for nestin. At 50 μg/mL, the reduction ranged from 23 to 42% for β-Tub III, from 30 to 50% for MAP-2, from 15 to 38% for NSE, and from 29 to 70% for nestin (Figure 8).

### 3.4. ATP Content Evaluation during Differentiation Process after Fe_3_O_4_NPs Exposure

A concentration-dependent reduction of the ATP content was observed in hNLCs exposed to 1–50 μg/mL Fe_3_O_4_NPs. In hNLCs, already after 2 days of transdifferentiation, the ATP content was reduced by 15% compared to control although at higher concentrations tested (25–50 μg/mL). During the different stages of differentiation process (i.e., from day 3 to 8 of transdifferentiation), hNLCs appeared susceptible to Fe_3_O_4_NPs exposure: ATP content decreased by 10–25% following single exposure to ≥5 μg/mL (Table 3). One μg/mL had no effect on ATP content.

### 3.5. Cytotoxicity Evaluation

A concentration-dependent reduction of the viable cells, evaluated by a loss cell membrane integrity, was observed in hNLCs exposed from 1 to 50 μg/mL to Fe_3_O_4_NPs during the different stages of differentiation: effects appeared early (on day 2 and 4) after exposure to higher concentrations (25–50 μg/mL), and persisted (Table 4). Lower concentrations (≥5 μg/mL) were also able to induce cell mortality (10–15%) starting from day 5 of differentiation. The lowest concentrations tested, namely 1 μg/mL, was devoid of any effect.

### 3.6. Evaluation of Caspase-3/7 Activity

**Figure 9:**
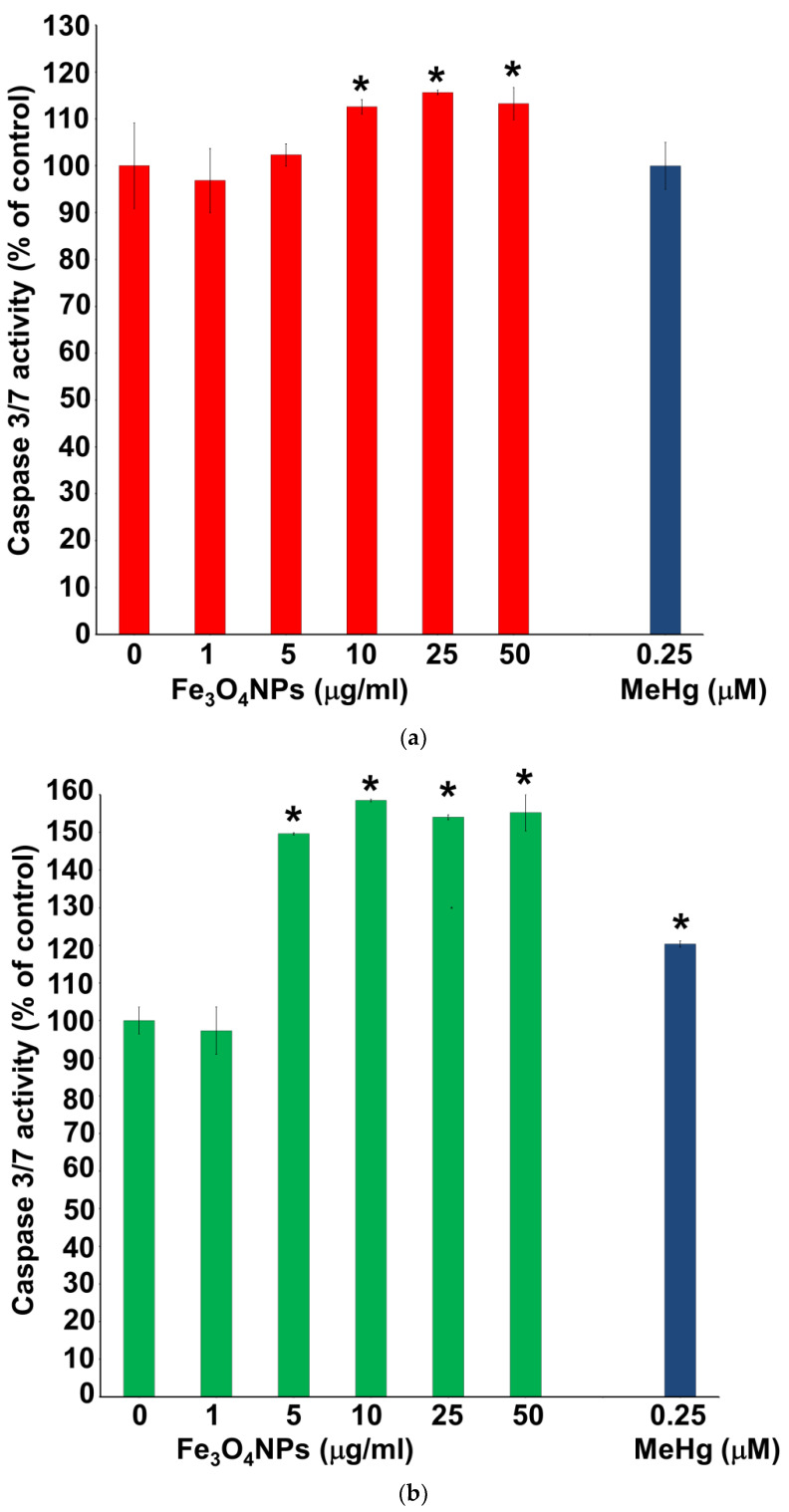
Caspase 3/7 activity evaluation in hNLCs treated with Fe_3_O_4_NPs. An increase of caspase 3/7 activity levels was observed in hNLCs starting from 10 μg/mL (+1.1-fold) at day 3 (**a**) and 5 μg/mL (+1.5-fold) at day 8 (**b**) of transdifferentiation. MeHg was used as positive control. Data represent the mean ± S.D. * *p* < 0.05. Statistical analysis by two-way ANOVA followed by Dunnett’s test.

Caspase-3/7 activity was assessed after Fe_3_O_4_NPs treatments in neuron-like cells (Figure 9a,b). In particular, the results obtained on hNLCs after 3 days of transdifferentiation showed that the levels of caspase-3/7 activity increased about 10% (compared to control) at 10 and 25 µg/mL (Figure 9a). After 8 days of transdifferentiation, a significant increase of caspase-3/7 activity of about 50% was also observed in hNLCs fully differentiated (day 8) starting from 5 µg/mL (Figure 9b).

## 4. Discussion

Our findings clearly indicated that CL-hMSCs differentiated into neuronal cells which could serve as novel in vitro model for neurotoxicity and developmental neurotoxicity studies. The differentiation process could be divided into four stages: undifferentiated (1 h after induction); early differentiated (2–3 days after induction); mid differentiated (4–5 days after induction); and fully differentiated (8 days) stage based on cell morphological transformation and the expression of several neuronal-specific markers such as β-Tub III, MAP-2, NSE, and lower nestin expression.

Specifically, the CL-hMSCs exhibited morphological changes over time (up to 8 days) when they were cultured in the neurogenic medium. Following 8 days, MSCs from human CL adopted a neural-like morphology ranging from spindle shaped to spherical with retractile cell bodies and long branching processes. This stepwise morphological differentiation was perfectly visible from day 2 to 8 under light microscopy throughout the differentiation process.

The levels of the different neuronal markers also characterized the different stages of the differentiation of CL-hMSCs into hNLCs. β-Tub III and MAP-2 were expressed during the neuronal differentiation of neural precursor cells and were detectable at early time of differentiation (from day 2) which further increased in fully differentiated cells (i.e., β-Tub III increased by 57% and MAP-2 by 95% at day 8 compared to day 2). The neuron-specific enolase, an established marker of neuronal differentiation behaved in a more fluctuant way but still was present immediately (at day 2). An early increased and later downregulated level of the early differentiation marker protein nestin (44% decrease from day 2 to day 8) further confirmed the differentiation process of MSCs into neuronal-like cells. These data are coherent with those of other studies reporting that human MSCs that differentiate into neuron-like cells exhibit increased characteristics of neuronal protein markers such as β-Tub III, MAP-2, and NSE [43,44,45,46,47]. Beta tubulins are one of two structural components that form microtubule network and β-Tub III is specifically localized to neurons. Beta-Tub III’s expression correlates with the earliest phases of neuronal differentiation and for this reason it has implications in neurogenesis, axon guidance and maintenance. MAP-2, a protein that belongs to the microtubule-associated protein family, is involved in microtubule assembly, and is primarily localized in the dendrites, implicating a role in determining and stabilizing dendritic morphology during neural development. In our model, MAP-2 highly increased during differentiation process. NSE also possesses neurotrophic properties on a wide spectrum of CNS neurons and is needed for neuronal cell survival.

Whether Fe_3_O_4_NPs interfere with the neuronal differentiation process of CL-hMSCs into hNLCs was determined after treating CL-hMSCs with different (from 10 to 50 μg/mL) concentrations of Fe_3_O_4_NPs (single exposure) at T0 of the neurogenic induction. Fe_3_O_4_NPs affected in a concentration dependent manner the neuronal differentiation with effects starting early (day 2) and persisting up to day 8. Monitoring the neuron-specific markers during different stages, the neuronal differentiation markers indicated a reduction in neuronal differentiation induction after nanoparticle exposure. In particular, β-Tub III, MAP-2 and NSE were downregulated (10–40%) starting from 25 μg/mL Fe_3_O_4_NPs and early since the effects were noticeable from day 2 of the induction process. Effects were exacerbated (expression decreased by 15–70%) at the higher concentrations (50 μg/mL) and persisted up to 8 days. Even nestin was downregulated during the induction process. Apparently, the NP-induced effects were observed both on specific neuronal differentiation markers (β-Tub III, MAP-2, NSE) as well as on neuronal precursor marker (nestin). Altogether these results demonstrate that NPs exposure induce effects on differentiation indicating a potential loss of function.

The possibility that NPs could interfere with cell differentiation has been addressed in previous studies, albeit with divergent outcomes. Hence, in our previous studies magnetite NPs did not impair the ability of human mesenchymal stem cells to differentiate into adipogenic and osteoblastic lineages [42]. Recent investigations, in other in vitro neuronal models, support the evidence that NPs may impact on neuronal differentiation, suggesting that these NPs could pose a developmental neurotoxicity hazard. In human SH-SY5Y cells during neuronal differentiation induction, a transient short exposure (24 h) to silica NPs reduced the neuronal differentiation markers, including MAP-2, and intracellular signaling pathways leading to a modification of neuronal differentiation, although the neurite outgrowth was not altered [48]. Inhibition of neuronal differentiation of neuronal stem cells (using C17.2 murine cell line) and interference with cytoskeletal organization were also observed after cerium oxide NPs exposure. In particular, the neuron specific β-Tub III expression was reduced after 50 μg/mL cerium oxide NPs exposure [49]. Moreover, morphological analysis indicated that Fe_2_O_3_NPs were able to reduce dramatically the ability of the mouse embryonic stem cells (Royan B1 cell line) to generate neuritis, and in particular the expression of β-Tub III was reduced in a dose-dependent manner starting from 10 μg/mL [50]. Different IONPs (i.e., Resovist, magnetoliposomes and Endorem), at 500 μg Fe/mL, have also been shown to affect the actin cytoskeleton and microtubule network architectures, focal adhesion formation and maturation, and protein expression levels in murine C17.2 neural progenitor cells. The extent of the effects correlated with the intracellular concentrations of the NPs [51].

Our data indicated that hNLCs allowed for efficient uptake of the Fe_3_O_4_NPs after mere incubation with the NPs simply suspended in the neuronal induction medium. In fact, a rapid accumulation of Fe_3_O_4_NPs was observed at day 3 and 8 of differentiation as evidenced by Perls’ Prussian blue staining which revealed Fe_3_O_4_NP particles uptake, as blue dots, inside the cytoplasm of hNLCs with accumulation in a concentration-dependent. Fe_3_O_4_NPs also sedimented and settled on the cell surface: they tended to be attached to either the cell membrane or the bottom of the plate.

Although the negative Zp and the wide size distribution values (ranging from about 1200 to 1500 nm) seem to be indicative of Fe_3_O_4_NPs agglomeration/aggregation into the medium as corroborated by brownish sediments visible under the microscope, apparently this phenomenon did not preclude the Fe_3_O_4_NPs accumulation in hNLCs, as also demonstrated by other authors [52,53].

In the present study, data from cytotoxicity assay which evaluated loss of cell plasma membrane integrity, a hallmark of necrotic cell death, revealed that the Fe_3_O_4_NPs were able to induce cell mortality in a concentration dependent manner during the different stages of differentiation (i.e., from day 2 to 8 of transdifferentiation). Effects on cell mortality appeared early (on day 2 and 4) after exposure to higher concentrations (25–50 μg/mL), and persisted. Even lower concentrations (≥5 μg/mL) were able to induce cell mortality (10–15%) starting from day 5 of differentiation.

Notably, cell viability, detected as ATP content, was also significantly reduced during the different stages of the differentiation process. hNLCs appeared susceptible to low Fe_3_O_4_NPs concentrations since ATP content decreased by 10–25% following single exposure to ≥5 μg/mL from day 3 to 8; and already after 2 days of transdifferentiation at the higher concentrations tested (25–50 μg/mL). Low Fe_3_O_4_NPs concentrations were able also to induce an increase of caspase 3/7 activity (10–50%). This apoptotic effect was detected at ≥10 μg/mL after 3 days of differentiation and at ≥5 μg/mL after 8 days.

Morphological evaluation by phase-contrast microscopy and Perls’ Prussian blue staining also evidenced a cell density decrease starting at Fe_3_O_4_NPs 25 μg/mL and on day 3 of the neurogenic induction. Apparently none of the concentrations tested induced any morphological alteration in hNLCs during the whole differentiation process (up to 8 days).

In summary, when Fe_3_O_4_NPs were added at the beginning (T0) of the neurogenic induction, cell number decrease (about 20%) and apoptosis increase occurred at ≥5 μg/mL and early (from 3 days), which thereafter persisted up to 8 days. Neuronal markers expression indicated a reduction in neuronal differentiation induction starting from 25 μg/mL and early (day 2 of differentiation) that persisted up to 8 days. No alterations of cell morphology were detected during neurogenic induction. The lowest concentrations tested, namely 1 μg/mL, was devoid of any effect.

Currently, in vitro neurotoxicity evaluation of Fe_3_O_4_NPs on human cell lines are limited [54]. Significant differences in cytotoxicity results were observed when compared data obtained on rodent cells with those obtained on human cells [55], suggesting the importance of the cell type and the species-specific system used to assess Fe_3_O_4_NPs toxicological profile. Notably, the findings of the present study further support the use of these human primary cells especially when considering the results obtained in cerebral cells from laboratory animals. In fact, recent studies evidenced a relative resistance of the rat astrocytes and neurons against short-term IONP exposure [56,57], although the experimental conditions [58,59] and the type of IONPs used [59,60,61] have been shown to influence the toxicity in terms of reactive oxygen species formation and delayed toxic effects in these types of CNS cells.

Altogether our findings proved that the critical concentrations of Fe_3_O_4_NPs capable to cause in vitro neurotoxicity were similar to those quantified in brain tissue (0.040–58 μg/g) and peripheral blood (350–375 μg/mL) of laboratory animals (i.e., mice, rats, rabbits) treated with IONPs, whose iron content was associated with cytotoxic effects (such as altered mitochondrial function, cell membrane damage, oxidative stress, inflammation, neuronal cell cycle induction resulting in apoptosis), increase of cerebral neurotransmitters content (such as dopamine and norepinephrine), and motor and memory deficits [62,63,64,65,66,67].

In humans, high iron levels appear to be associated with many types of neurodegenerative disease [68,69,70,71,72]. It has been observed that the surplus of iron in neurodegenerative tissues appears as magnetic Fe_3_O_4_ [73], hypothesis sustained by elevated concentration of Fe_3_O_4_ detected in samples of Alzheimer’s disease tissue [74]. Recently, brain magnetite nanospheres were identified in human subjects and were consistent with an external, rather than an endogenous, source [35,36]. Their presence demonstrates that externally sourced iron-bearing NPs can reach the brain, where they result in hazard to human health.

Despite the wide IONP utilization in environmental and occupational setting and numerous IONP applications being explored for diagnostic and therapeutic applications, insufficient information is available on their potential toxicity and in particular neurotoxicity. The volume of studies on the toxicity of NPs in the reproductive system of animals is increasing, but the field is effectively still in its preliminary stages, in particular regarding to magnetite NPs. Considering potential release of the magnetite NPs into the environment and accumulation in human tissues there is an urgent need to figure out the potential to be dangerous in the adult as well as in the fetal brain. Studies using animal models have demonstrated that different types of NPs (i.e., Au, TiO_2_, SiO_2_, carbon, and QDs) are able to cross the placenta and transfer to vulnerable fetus causing variable toxic effects on fetal brain, nerve development and future fertility [14]. In particular, to metallic NPs, some studies have documented that exposure during pregnancy can affect fetal brain development [75,76,77], and underlined that much attention should be paid to the role of apoptosis in developmental neurotoxicity induced by NPs [12]. In addition to TiO_2_NPs and silver NPs that were most widely studied, other metallic nanomaterials including NPs of iron oxide among others should be investigated to understand the pivotal role of apoptosis in the neurotoxicity of NPs and to determine the consequences of IONP exposure on embryo and fetal development [12,54].

In this context, according to the development of new and innovative approaches for assessing emerging contaminants safety (including nanomaterials), the stem cell-derived in vitro model may offer more suitable platforms for the studying of the toxicity, and the hMSC capacity to transdifferentiate into hNLCs can further encourage the use of the latter for the screening assessment of neuronal toxicity of these compounds in humans. The approach described in this study follows the most updated guidance on using human cell-based systems to investigate human diseases and toxic effects induced by xenobiotics. Notably, human MSCs generated from umbilical cord are increasingly under investigation as a promising source for, not only a stem cell-based therapy, but even in vitro model for toxicity screening of different compounds (i.e., drugs, chemicals, nanoparticles), either in studying target organ toxicity or developmental toxicity. Researchers are now putting a lot of effort into obtaining differentiated cultures from human stem cells, as these can provide an unlimited supply of “natural” human cells. For example, the group of Buzanska [25] has successfully established a human neural stem cell line generated from umbilical cord blood (HUCB-NSC) demonstrating the sensitivity at different phases of development to a panel of compounds including neurotoxicants. HUCB-NSCs at early developmental stage displayed more susceptibility to neurotoxicants. The authors concluded that these HUCB-NSCs may serve as a reliable test (human in vitro) model for DNT [25]. More recently, a human embryonic stem cell (hESC)-derived 3-dimensional (3-D) in vitro model have been developed for testing potential developmental neurotoxicants. The data have suggested that this 3-D hESC-derived model could be used to test for Nano-DNT [78].

In line with this approach, the present study demonstrated that human umbilical CL-hMSCs easily differentiated into neuronal-like cells. The human umbilical CL-hMSCs are versatile stem cells easily to be obtained and expanded from healthy human subjects without any ethical restriction. These cells possess a strong proliferative capability (self-renewal), long-term proliferation and stable amplification in vitro. In addition, the present study demonstrated that CL-hMSCs easily transdifferentiated into neuronal-like cells of different stages which may serve as novel in vitro system also for developmental neurotoxicity studies. These intrinsic properties of CL-MSCs make them an excellent tool for establishing in vitro models of neurotoxicity and development NT.

## 5. Conclusions

Altogether the present in vitro findings add value to the relevance of using novel in vitro human cell-based models in neurotoxicology and, in particular, for the identification of the cytotoxicity effects induced by Fe_3_O_4_NPs exposure. Predictive toxicology is primarily focused on human derived systems, as those proposed in this research study, and human outcomes. Predictivity for the in vivo situation in humans with modern in vitro methods is continuously growing. In view of the limited access to human tissue, stem cells are likely to gain more and more importance as a replacement for primary human cells. In this context, the proposed new cell-based models derived from human mesenchymal stem cells associated with a panel of complementary tests, will provide new models/methods for the initial hazard identification for xenobiotic risk assessment as a screening strategy to define the toxicity of emerging toxic compounds in terms of mechanistic understanding of the cellular responses. This in vitro system allows different stages of hNLCs to be cultured, exposed to NPs/chemicals, and analyzed for different endpoints at an early or a later developmental stage.

## Figures and Tables

**Table 1:** Physico-chemical properties of Fe_3_O_4_NPs stock suspension.

Fe_3_O_4_NPs Stock Suspension	
**Diameter (TEM) (nm)**	20.3 ± 5
**Coefficient of Variation (%)**	24.6
**Surface Area (TEM) (m^2^/g)**	50.2
**Mass Concentration (mg/mL)**	20.3
**Hydrodynamic Diameter (nm)**	42
**Zeta Potential (mV)**	−51
**pH Solution**	7.4

TEM, transmission electron microscopy.

**Table 2:** Physico-chemical properties of Fe_3_O_4_NPs suspension in neurogenic medium.

**10 μg/mL Fe_3_O_4_NPs**				
	**Mean diameter (nm)**	**Zp** **(mV)**	**PdI**	**pH**
**Time**				
**30 min**	1213 (23.5)	−9.5 (0.59)	0.264 (0.01)	8.09
				
**24 h**	1321 (50.9)	−8.9 (0.68)	0.305 (0.09)	7.78
				
**48 h**	1552 (158.0)	−9.40 (034)	0.230 (0.05)	7.65
**25 μg/mL Fe_3_O_4_NPs**				
	**Mean diameter (nm)**	**Zp** **(mV)**	**PdI**	**pH**
**Time**				
**30 min**	1368 (10.0)	−11.30 (0.59)	0.264 (0.01)	8.09
				
**24 h**	1405 (38.7)	−10.20 (0.32)	0.229 (0.01)	7.64
				
**48 h**	1450 (36.7)	−10.30 (0.25)	0.227 (0.03)	7.65

Zp, zeta potential; PdI, polydispersity index. Data are expressed as the mean ± (S.D.) of three measurements (data adapted from De Simone et al. [30]).

**Table 3:** ATP content evaluation in hNLCs plus Fe_3_O_4_NPs (1–50 μg/mL) over time.

Fe_3_O_4_NP (μ/mL)						
hNLCs	0	1	5	10	25	50
**day 2**	100 ± 7.16	109.60 ± 3.40	96.70 ± 1.18	104.61 ± 0.94	85.96 ± 2.35 *	84.68 ± 2.61 *
**day 3**	100 ± 3.74	106.87 ± 4.87	92.12 ± 2.89 *	88.50 ± 6.52 *	78.70 ± 4.50 *	79.91 ± 4.40 *
**day 4**	100 ± 5.22	95.89 ± 2.85	89.18 ± 4.77 *	93.71 ± 0.61 *	87.38 ± 1.69 *	91.07 ± 3.16 *
**day 5**	100 ± 3.71	102.34 ± 1.21	84.12 ± 3.98 *	87.27 ± 4.38 *	83.89 ± 3.65 *	84.07 ± 5.10 *
**day 8**	100 ± 6.20	111.81 ± 3.04	90.95 ± 2.09 *	85.90 ± 4.48 *	75.49 ± 3.66 *	74.46 ± 1.62 *

Data are expressed as percentage of viable cells (% of each control) and represent the mean ± S.D. * *p* < 0.05, statistical analysis by two-way ANOVA followed by Dunnett’s test.

**Table 4:** Cell viability evaluation in hNLCs plus Fe_3_O_4_NPs (1–50 μg/mL) over time.

Fe_3_O_4_NP (μ/mL)						
hNLCs	0	1	5	10	25	50
**day 2**	100 ± 4.20	99.52 ± 1.38	97.33 ± 2.18	98.97 ± 1.10	96.37 ± 1.42	90.79 ± 5.88 *
**day 3**	100 ± 6.58	98.46 ± 12.93	96.13 ± 7.08	94.39 ± 5.11	91.14 ± 2.15	90.75 ± 0.84 *
**day 4**	100 ± 3.76	101.39 ± 1.42	95.51 ± 2.07	94.45 ± 1.95	89.58 ± 0.66 *	89.56 ± 1.61 *
**day 5**	100 ± 2.41	92.90 ± 3.94	90.34 ± 4.35 *	90.11 ± 1.94 *	81.86 ± 2.51 *	81.19 ± 0.87 *
**day 8**	100 ± 4.48	92.92 ± 0.01	89.61 ± 4.04 *	84.01 ± 3.13 *	85.95 ± 2.56 *	78.64 ± 2.03 *

Data are expressed as percentage of viable cells (% of each control) and represent the mean ± S.D. * *p* < 0.05, statistical analysis by two-way ANOVA followed by Dunnett’s test.

## Supplementary Materials

The following are available online at https://www.mdpi.com/2079-4991/10/8/1607/s1, Figure S1: Morphological analysis of hNLCs cultured with increasing concentration of Fe_3_O_4_NPs, Figure S2: Neuronal marker protein expressions in hNLCs cultured with Fe_3_O_4_NPs (10–50 μg/mL).
