# Supplementary material for: Developmental Neurotoxicity Screening for Nanoparticles Using Neuron-Like Cells of Human Umbilical Cord Mesenchymal Stem Cells: Example with Magnetite Nanoparticles
Source: Nanomaterials (Basel). 2020 Aug 15;10(8):1607. doi: 10.3390/nano10081607 (PMC7466682; doi:10.3390/nano10081607)
Supplement: Supplementary file 1 [file nanomaterials-10-01607-s001.pdf]

Developmental Neurotoxicity Screening for Nanoparticles Using Neuron-Like Cells of Human Umbilical *Cord Mesenchymal Stem Cells:* Example with Magnetite Nanoparticles

Teresa Coccini [1,*], Patrizia Pignatti [2], Arsenio Spinillo [3] and Uliana De Simone [1]

1 Toxicology Unit, Laboratory of Clinical and Experimental Toxicology, Istituti Clinici Scientifici Maugeri IRCCS, Via Maugeri 10, 27100 Pavia, Italy; uliana.desimone@icsmaugeri.it
2 Allergy and Immunology Unit, Istituti Clinici Scientifici Maugeri IRCCS, Via Maugeri 10, 27100 Pavia, Italy; patrizia.pignatti@icsmaugeri.it
3 Department of Obstetrics and Gynecology, Fondazione IRCCS Policlinico San Matteo and University of Pavia, 27100 Pavia, Italy; spinillo@smatteo.pv.it
* Correspondence: teresa.coccini@icsmaugeri.it; Tel.: +39-0382-592416

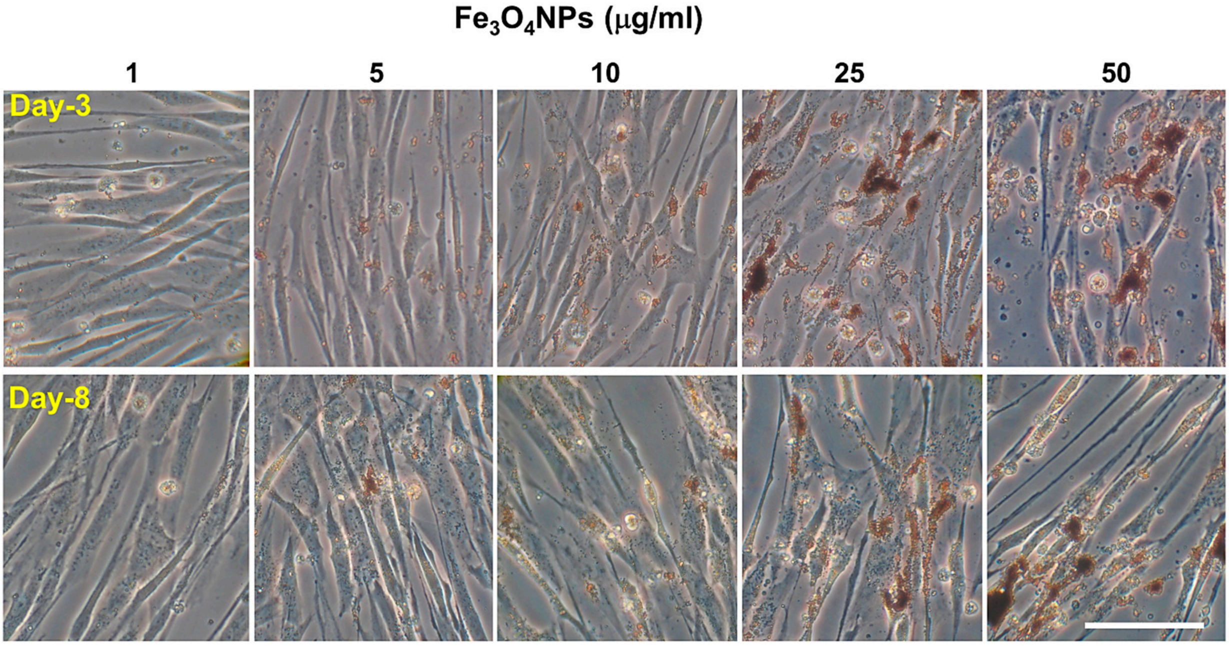

Figure 1. Morphological analysis of hNLCs cultured with increasing concentration of Fe_3O_4NPs. Representative micrographs, by phase-contrast microscopy, of hNLCs after 3 and 8 days of culture in neurogenic medium with Fe_3O_4NPs (from 1 to 50 µg/ml). No morphological changes were induced by Fe_3O_4NPs exposure in hNLCs at day 3 and 8 of transdifferentiation. However, a cell density decrease was observed from 25 µg/ml Fe_3O_4NPs at both time points considered. Brownish aggregates/agglomerates of Fe_3O_4NPs were also observed in culture medium. Scale bar: 100 µm.

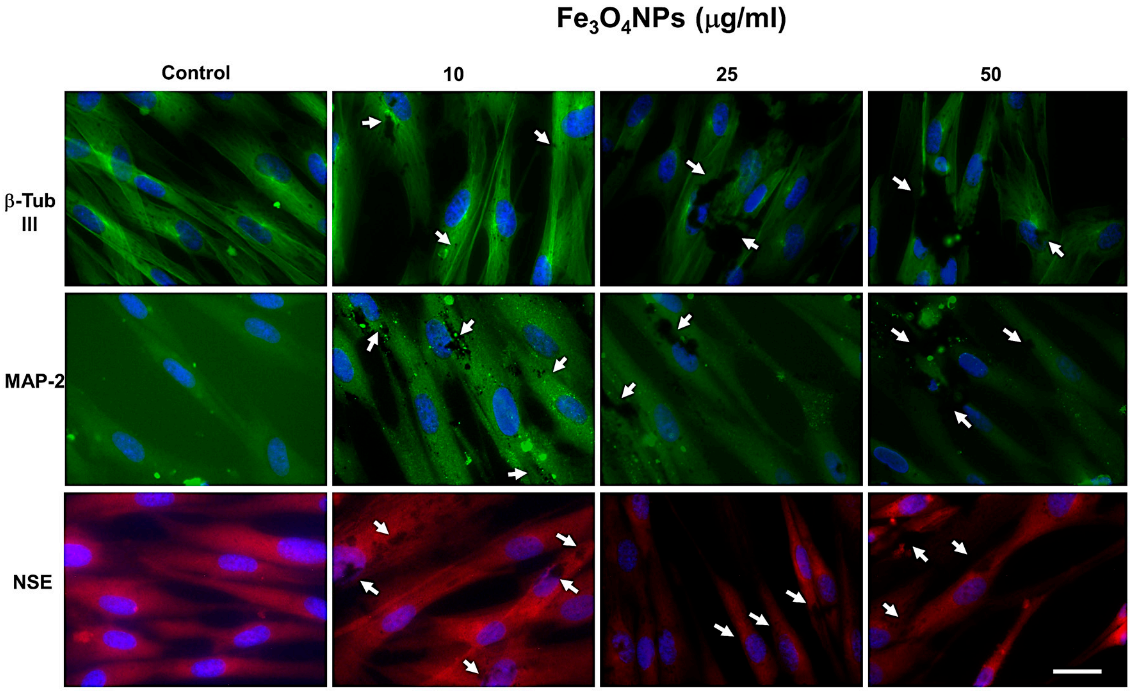

(**a**)

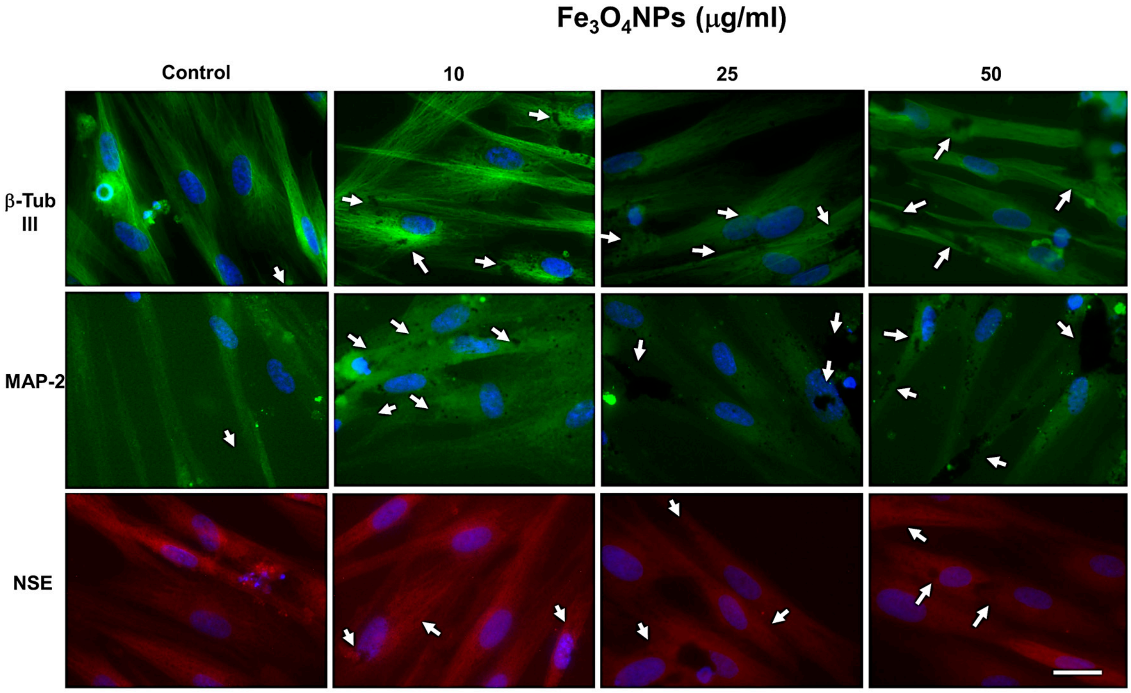

(**b**)

Figure S2. Neuronal marker protein expressions in hNLCs cultured with Fe_3O_4NPs (10-50 μg/ml). Representative fluorescence merged images of hNLCs at day 3 (**a**) and day 8 (**b**) of transdifferentiation. Fe_3O_4NPs affected the expression of neuronal proteins such as β-Tub III (green fluorescence), MAP-2 (green fluorescence) and NSE (red fluorescence): a light fluorescence intensity decrease of the neuron markers was observed starting from ≥ 25 μg/ml. Black spots of Fe_3O_4NPs (intracellularly and on the cell membrane) were also visible. Nuclei were stained with Hoechst 33258. White arrows indicat Fe_3O_4NPs (dark spots). Scale bar: 100 μm.
